# Stem Cell-Derived Exosome as Potential Therapeutics for Microbial Diseases

**DOI:** 10.3389/fmicb.2021.786111

**Published:** 2022-02-14

**Authors:** Somayeh Keshtkar, Maryam Kaviani, Saeede Soleimanian, Negar Azarpira, Zahra Asvar, Sara Pakbaz

**Affiliations:** ^1^Molecular Dermatology Research Center, Shiraz University of Medical Sciences, Shiraz, Iran; ^2^Transplant Research Center, Shiraz University of Medical Sciences, Shiraz, Iran; ^3^Nanotechnology School of Advanced Medical Sciences and Technologies, Shiraz University of Medical Sciences, Shiraz, Iran; ^4^Department of Laboratory Medicine and Pathobiology, Faculty of Medicine, University of Toronto, Toronto, ON, Canada; ^5^Department of Pathology, University Health Network, Toronto, ON, Canada

**Keywords:** exosome, stem cell, anti-microbial, pathogen, therapy

## Abstract

Exosomes, as the smallest extracellular vesicles that carry a cargo of nucleic acids, lipids, and proteins and mediate intercellular communication, have attracted much attention in diagnosis and treatment in the field of medicine. The contents of exosomes vary depending on the cell type and physiological conditions. Among exosomes derived from several cell types, stem cell-derived exosomes (stem cell-Exo) are increasingly being explored due to their immunomodulatory properties, regenerative capacity, anti-inflammatory and anti-microbial functions. Administration of stem cell-Exo, as a cell-free therapy for various diseases, has gained great promise. Indeed, the advantages of exosomes secreted from stem cells outweigh those of their parent cells owing to their small size, high stability, less immunogenicity, no risk of tumorigenesis, and easier condition for storage. Recently, the use of stem cell-Exo has been proposed in the field of microbial diseases. Pathogens including bacteria, viruses, fungi, and parasites can cause various diseases in humans with acute and chronic complications, sometimes resulting in mortality. On the other hand, treatments based on antibiotics and other chemical compounds have many side effects and the strains become resistant to drugs in some cases. Hence, this review aimed to highlight the effect of stem cell-derived extracellular vesicles including stem cell-Exo on microbial diseases. Although most published studies are preclinical, the avenue of clinical application of stem cell-Exo is under way to reach clinical applications. The challenges ahead of this cell-free treatment that might be applied as a therapeutic alternative to stem cells for translation from bench to bed were emphasized, as well.

## Graphical Abstract

Summary of the antimicrobial effect of stem cell-derived exosomes on bacteria, viral, fungal, and parasitic diseases.

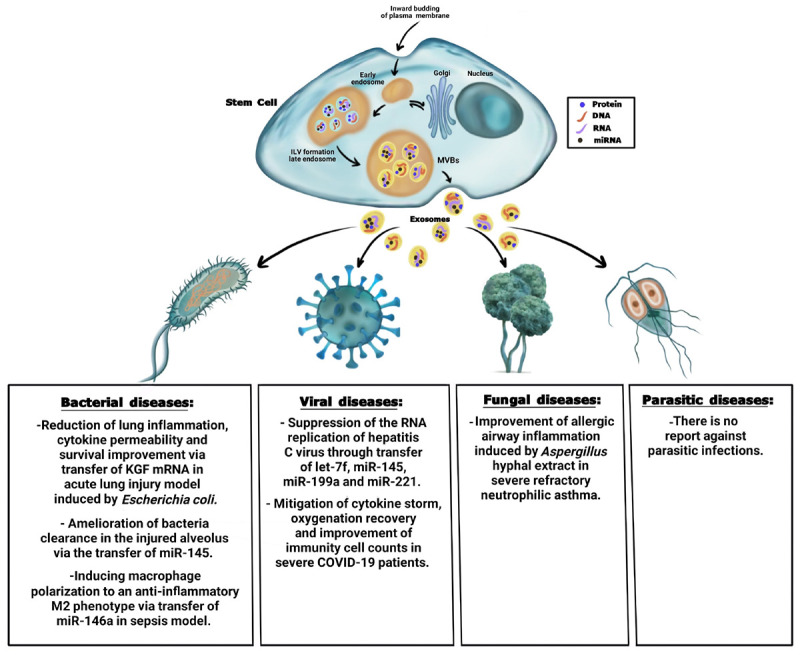

## Introduction

**FIGURE 1:**
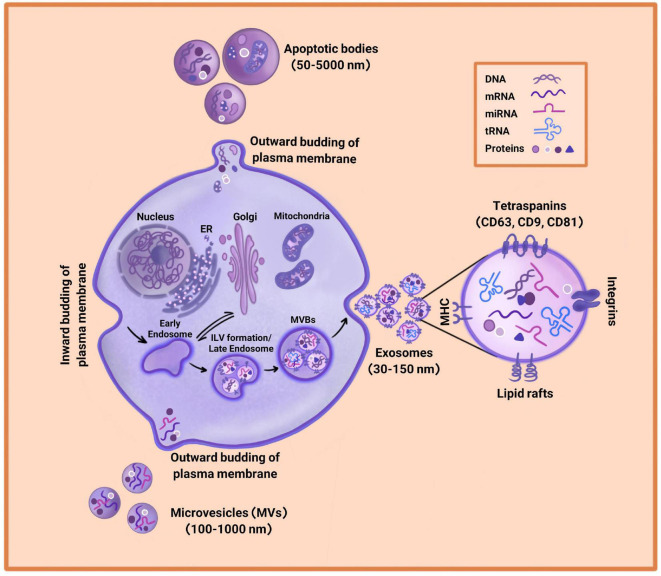
Extracellular Vesicles (EVs) biogenesis. EVs consist of exosomes, microvesicles, and apoptotic bodies. Exosomes arise through the inward budding of plasma membrane and are the smallest in diameter (30–150 nm). Microvesicles are larger in diameter (100–1,000 nm) and are driven through an outward budding of the plasma membrane. Apoptotic bodies range from 50 to 5,000 nm in diameter and are generated by outward budding of the plasma membrane from dying cells. Due to different biogenesis mechanisms, the compositions of exosomes, microvesicles, and apoptotic bodies are varied.

Almost all physiological and metabolic processes depend on cell-to-cell communication. Extracellular Vesicles (EVs) are one of the most important mediators of intercellular communication (Wang et al., 2018; Larabi et al., 2020), which include a collection of vesicles enclosed in a phospholipid bilayer membrane and released by various cells into the extracellular space. The process of EVs release is evolutionary conserved and occurs in both prokaryotes and eukaryotic cells. EVs were first identified by Dr. Ross Johnstone in 1983 during reticulocyte maturation. Before that, EVs were considered “garbage bags,” but they turned out to play an important role in intercellular communication by transferring different kinds of protein, nucleic acid, and lipid within an organism or between species (Keshtkar et al., 2018; Larabi et al., 2020). The International Society of Extra Cellular Vesicles (ISEV) has classified EVs into three groups of exosomes, microvesicles, and apoptotic bodies based on their size, biogenesis, release routs, cargos, and function (Figure 1). It has been shown that all types of cell are able to release EVs, which are observed in almost all body fluids such as blood, normal urine, breast milk, bronchial lavage fluid, saliva, cerebrospinal fluid, amniotic fluid, and synovial fluid (Keshtkar et al., 2018; Zhao et al., 2020).

Extracellular Vesicles, particularly exosomes, are released by cells during normal physiological and pathological conditions (Yuana et al., 2013). Production of EVs can be induced by various processes such as oxidative stress, hypoxia, senescence, inflammation, and infection (Keshtkar et al., 2018; Zhao et al., 2020). The number of released EVs depends on the physiological state of cell production and its microenvironment. The unique properties of EVs in delivering their active cargos to neighbor or distant cells have attracted much attention for the therapeutic application of these particles (Abreu et al., 2016; Zhang et al., 2019). In the following sections, three groups of EVs are reviewed, focusing on exosomes and exosomes derived from stem cells.

## Apoptotic Bodies

Apoptotic bodies are the biggest vesicles whose diameters range from 50 to 5,000 nm and are released from dying cells (Doyle and Wang, 2019). The process of apoptotic body formation includes cell contracts and enhancement of hydrostatic pressure, leading to outward budding or fragmentation of plasma membrane from cytoskeleton in dying cells (Figure 1). It has been reported that apoptotic bodies carry chromatin, intact organelles, and glycosylated proteins such as histones and Heat Shock Protein (HSP)-60. The markers of apoptotic bodies include Annexin V, DNA fragments, and histones. However, their contents can be different depending on the cell type from which they are released (Doyle and Wang, 2019; Mohan et al., 2020; Rezaie et al., 2021).

## Microvesicles

Microvesicles (MVs) are 100–1,000 nm EVs released from a verity of living cells into the extracellular space (Figure 1). MVs biogenesis is driven *via* direct outward budding of plasma membrane in the presence of cytoskeleton agents such as microtubules, actin, kinesins, myosins, and tethering factors (Doyle and Wang, 2019; Rezaie et al., 2021). These vesicles isolate by ultracentrifugation at 10,000–20,000 × *g*. Since MVs are separated by the budding of plasma membrane, their composition mainly includes plasma membrane-associated proteins such as tetraspanins along with cytosolic proteins, cytoskeletal proteins, and HSPs. However, its compounds are not limited to proteins and contain lipids, mRNAs, and microRNAs. Moreover, MVs are one of the mediators of cell-to-cell communication between neighbor and distant cells, which interact through a specific ligand-receptor. MVs are able to transfer their contents to recipient cells and change the functionality of target cells based on physiological and pathological environmental conditions (Keshtkar et al., 2018; Doyle and Wang, 2019).

## Exosomes

Exosomes are nano-vesicles with sizes ranging from 30 to 150 nm in diameter that sediment between 70,000 and 200,000 × *g*. Exosomes arise through a specific biogenesis pathway including the inward budding of plasma membrane that forms the early endosome (Figure 1). Then, the early endosome matures into late endosome with the accumulation of Intraluminal Vesicles (ILVs) in their lumen. The process of ILVs formation is mediated by the Endosomal Sorting Complex Required for Transport (ESCRT) or by ESCRT-independent mechanisms including tetraspanins or lipids such as ceramides. Since the late endosome contains ILVs, it is called Multivesicular Bodies (MVBs) (Yuana et al., 2013; Salimi et al., 2020). Finally, MVBs fuse with plasma membrane and generate exosomes (Larabi et al., 2020). After releasing into the extracellular space, exosomes are recognized by recipient cells through adhesion factors such as integrin followed by endocytic uptake. However, some exosomes directly fuse with the plasma membrane or interact with the lipid-ligand receptor and transmit their cargos (Keshtkar et al., 2018; Shi et al., 2021). Depending on the distance of the target cell, exosomes for distant cells may be absorbed through the paracrine or the endocrine pathway (Keshtkar et al., 2018).

The importance of exosomes goes back to their contents since they contain a valuable shipment of proteins, lipids, and metabolites as well as a set of nucleic acids consisting of microRNA, tRNA fragments, mRNAs, small RNA transcripts, and RNA-protein complexes (Wang et al., 2018; Larabi et al., 2020). Exosomes also carry chromosomal and mitochondrial DNA. It has turned out that these nucleic acids are essential in cell signaling transduction and regulation of biological function. Nucleic acids are functionally active when entering recipient cells. Although the composition of exosomes depends on the origin of the donor cell, there are multiple conserved proteins that are considered the specific markers of exosomes. The tetraspanin family of proteins (including CD9, CD63, and CD81) are the most important conserved proteins, which assist the connection of inside the cell to the outside environment. Apart from tetraspanin, other important cell adhesion molecules include integrins and antigen presentation molecules (MHC) (Nikfarjam et al., 2020). HSP70 and HSP90 are also known as the top exosomal markers involved in membrane remodeling *via* protein folding regulation and transformation (Shi et al., 2021). Alix, TSG101, and GTPases are other specific markers of exosomes. Exosomes are surrounded by a lipid bilayer membrane that preserves them from degradation by the immune system and separation from body fluids. Exosomes enclosed in the phospholipid membrane include high levels of cholesterol, sphingomyelin, ceramide, and lipid-rafts (Doyle and Wang, 2019; Larabi et al., 2020). These lipids are in fact the characteristic of the cellular source releasing exosomes. After isolation, exosomes are stable and can be stored at −80°C for a long time without losing their functionality, because of their biolayer lipid membrane.

Studies have shown that exosomes possess immunomodulatory potentials, one of which being communication between antigen-presenting and recipient cells. Exosomes also contain cytokines with antimicrobial properties and innate response signaling molecules that are important in response to viral and bacterial infections. The exosomal composition depends mainly on the source of donor cells, epigenetic changes, and physiological and pathological microenvironment conditions. Hence, exosomes have vital roles in intracellular communication and immune modulation in different physiological and pathological conditions (Doyle and Wang, 2019). Due to the broad biological functions of exosomes including maintaining homeostasis and transferring molecules between cells, these vesicles have attracted much attention in medical research, with a focus on the therapeutic application of exosomes in the last two decades.

## Stem Cell-Derived Exosomes

It has been reported that exosomes are released by all kinds of cell including stem cells and immune cells that can enter body fluids including blood, saliva, amniotic fluid, urine, milk, cerebrospinal fluid, ascites, and semen (Wang et al., 2018) and move toward target cells. Among exosomes derived from several stem cell types, Mesenchymal Stem Cell-derived Exosomes (MSC-Exo) have received much attention due to their immunomodulatory, regenerative, and anti-inflammatory functions. MSCs exert immunoregulatory and tissue repair functions due to secreting paracrine factors including exosomes and MVs (Keshtkar et al., 2018; Zhao et al., 2020). MSC-Exo are involved in cellular processes including proliferation, transcription, migration, and differentiation. MSC-Exo also help the stimulation of angiogenesis, suppression of fibrosis, increase of neuronal survival and differentiation, induction of extracellular matrix remodeling, inhibition of local inflammation response, and adjustment of immune cells’ activities (Zhao et al., 2020).

Extensive body of evidence has demonstrated that MSC-Exo mimic the beneficial effects of parent MSCs in animal models of various human diseases including cardiovascular, kidney, liver, lung, and neurodegenerative diseases, and other diseases (Keshtkar et al., 2018, 2020; Zhao et al., 2020). MSC-Exo were first separated in 2010 and decreased the infarct size in a mouse model of myocardial ischemia/reperfusion injury. The results of microarray analysis indicated that about 98% of miRNAs in stem cells were in exosomes and MVs (Hassanzadeh et al., 2021; Rezaie et al., 2021).

In addition to the aforementioned beneficial effects, stem cell-Exo present anti-microbial properties like parent cells. pathogens including bacteria, viruses, fungi, and parasites can cause various diseases in humans with acute and chronic complications, sometimes resulting in mortality. Moreover, the rising incidence of emerging infectious agents is alarming. On the other hand, treatments based on antibiotics and other chemical compounds have many side effects and the strains become resistant to drugs in some cases. Hence, exploring novel treatment approaches is always a necessity. Recently, various studies have presented the anti-microbial effects of stem cell-Exo in preclinical and few clinical trials. This review highlights the recent studies exploring the therapeutic potential of all kinds of stem cell-Exo along with immune-derived exosomes to combat with microbial infections and complications.

## The Application of Stem Cells Therapy in Microbial Diseases

To date, stem cell therapy has been promising in tissue and immune disorders. Successful attempts have been made mainly MSCs in the treatment of infectious diseases and controlling their complications. This part aims to summarize the advances in this field.

The anti-bacterial effect of MSCs has been investigated in various studies (Krasnodembskaya et al., 2010; Sung et al., 2016; Liu et al., 2017; Chow et al., 2020). Accordingly, these cells exert their effect through direct bacterial killing or indirectly by modulating the acute phase of the immune response (Pierce and Kurata, 2021). MSCs express various kinds of anti-microbial peptide and protein (AMPs), four of which are well known due to anti-bacterial properties including cathelicidin LL-37 (Krasnodembskaya et al., 2010), β-defensin-2 (BD-2) (Sutton et al., 2016), hepcidin (Alcayaga-Miranda et al., 2015), and Lipocalin-2 (Lcn2) (Gupta et al., 2012). Recent studies have suggested that MSCs improve bacterial clearance in preclinical models through the AMPs. Therefore, MSCs can augment the innate immune response against bacteria (Alcayaga-Miranda et al., 2017). Yagi et al. (2020) assessed the anti-microbial activity of human Adipose-Derived MSCs (AD-MSCs) on *Staphylococcus aureus*. The findings indicated that human AD-MSCs conditioned medium significantly prevented the growth of *S. aureus.* The results also demonstrated that cathelicidin LL-37 played an important role in the anti-microbial activity of AD-MSCs (Yagi et al., 2020). A previous study also showed that the anti-microbial activity of BM-MSCs against the growth of Gram-negative (*Escherichia coli* and *Pseudomonas aeruginosa*) and Gram-positive (*S. aureus*) bacteria was mediated by LL-37 (Krasnodembskaya et al., 2010). On the other hand, human umbilical cord blood-derived MSCs attenuated acute lung injury due to *E. coli* infection in mice. The results demonstrated that MSCs secreted BD-2 through the TLR-4 signaling pathway and mediated the anti-microbial effects (Rezaie et al., 2021). Moreover, menstrual-derived MSCs were responsible for bacterial clearance by the secretion of hepcidin in synergy with antibiotics in sepsis (Alcayaga-Miranda et al., 2015). MSCs also exerted anti-bacterial activity through the secretion of growth factors, especially Keratinocyte Growth Factor (KGF) (Lee et al., 2013). In the research performed by Lee et al. (2013) BM-MSCs improved alveolar fluid bacterial clearance and mitigated inflammation in an *E. coli* infection model in an *ex vivo* perfused human lung (Lee et al., 2013).

The application of stem cells for the treatment of viral infections has started recently. Studies on the use of stem cells in the treatment of viral diseases are mainly related to MSCs. There are several clinical trials on the use of MSCs in the treatment of viral infections (Sleem and Saleh, 2020). These trials have been mainly focused on COVID-19 (Leng et al., 2020; Zhang et al., 2020), Human Immunodeficiency Virus (HIV) (Zhang et al., 2013), and hepatitis B virus (Lin et al., 2017; Chen B et al., 2018a). During the COVID-19 crisis, stem cells were introduced as the most promising treatment option (Chrzanowski et al., 2020). A recent clinical trial also indicated that MSCs improved COVID-19 patients’ outcomes. This improvement was dependent on the inhibition of the over-activation of the immune system. In fact, MSCs therapy decreased C-reactive protein and increased peripheral lymphocytes and IL-10 (Leng et al., 2020). Furthermore, Zhang et al. designed a pilot study to evaluate the responses of difficult-to-treat HIV-1-infected patients to human umbilical cord MSCs therapy. This treatment resulted in an increase in circulating naive and central memory CD4 T-cell counts and a decrease in systemic immune activation and inflammation. Furthermore, HIV-1-specific IFN-γ and IL-2 production was restored in immune non-responders (Zhang et al., 2013). Moreover, peripheral infusion of Bone Marrow–derived MSCs (BM-MSCs) significantly improved the survival rate in patients with hepatitis B virus–related acute-on-chronic liver failure because of recovering the liver function and reducing the incidence of severe infections (Lin et al., 2017).

The efficiency of stem cells in the treatment of parasitic infections has been reported in animal models (Zhang et al., 2014). However, there are limited therapeutic methods in parasitic infections, and drug resistance is a challenging issue in long-term drug administration (Ouellette, 2001; Montazeri et al., 2018; Ertabaklar et al., 2020). Previous studies indicated that stem cells played an important role in the treatment or control of schistosomiasis (Miranda et al., 2020), malaria (Souza et al., 2015), Chagas disease (Silva et al., 2014), and hydatid cyst (Abo-Aziza et al., 2019). Recently, Miranda et al. (2020) reported that AD-MSCs could decrease liver damage in schistosomiasis through controlling the granulomatous reaction. On the other hand, stem cells have been introduced as a new therapeutic option for malaria (Wang et al., 2015a). In this regard, BM-MSCs reduced mortality in infected mice. The results also revealed the reduction of parasitemia and morphological and functional improvement in vital organs (Souza et al., 2015). Furthermore, the effect of MSCs on protective immune responses was proposed in malaria-infected mice. Based on the results, these cells increased the production of IL-12, suppressed IL-10 production, and reduced regulatory T cells (Thakur et al., 2013). Moreover, the potentiality of stem cells was confirmed against myocarditis in Chagas disease. The findings showed that receiving cardiac MSCs attenuated myocarditis in a model of chronic Chagasic cardiomyopathy, but did not decrease fibrosis (Silva et al., 2014). Additionally, the combination of BM-MSCs transplantation with albendazole was effective in the modulation of humeral and cell-mediated immune responses against hydatid cyst antigens in experimentally infected rats (Abo-Aziza et al., 2019).

There are limited reports on the anti-fungal activity of stem cells. In a mouse model of severe refractory neutrophilic asthma, administration of BM-MSCs mitigated inflammation and improved the diseases induced by *Aspergillus via* Th17 inhibition. Moreover, a recent study indicated the anti-fungal activity of human uterine cervical stem cells conditioned medium against different species of Candida (Schneider et al., 2018).

## The Application of Stem Cell-Derived Exosomes in Microbial Diseases

An extensive body of evidence has indicated that a variety of cells including stem cells release exosomes and exert therapeutic properties in viral, bacterial, parasitic, and fungal infections, which will be discussed below.

## The Application of Stem Cell-Derived Exosomes in Bacterial Diseases

Bacterial infections are a major public health problem, and the enhanced antibiotic resistance of bacteria requires finding new therapeutic options (Monsarrat et al., 2019). The anti-bacterial effects of stem cell-Exo and MVs have been investigated in different bacterial diseases, especially respiratory failure (Zhu et al., 2014). One of the main causes of respiratory failure is Acute Lung Injury (ALI) that is mainly induced by bacterial pneumonia. Studies have demonstrated that stem cell-Exo have the potential to reduce the severity of bacterial pneumonia. However, little is known regarding the underlying mechanisms of their anti-microbial activity. Zhu et al. (2014) disclosed that BM-MSC-derived MVs were as effective as their parent cells in improving survival, restoring lung protein permeability, and reducing inflammation in an *E. coli* endotoxin-induced ALI mouse model. In fact, the administration of MVs decreased extravascular lung water, total protein level, and influx of neutrophils in Bronchoalveolar Lavage Fluid (BALF), indicating mitigation in pulmonary edema, lung protein permeability, and inflammation. Moreover, the anti-bacterial effect of MVs was in part through the transfer of KGF mRNA into the injured alveolus, which was eliminated after the administration of MVs derived from KGF siRNA-pre-treatment of BM-MSCs. KGF was known as a paracrine factor secreted by human MSCs and was previously revealed to restore alveolar fluid clearance (Abreu et al., 2016). This suggested the direct anti-bacterial activity of vesicles inherited from parent cells. In the same line, Monsel et al. (2015) reported that the administration of human MSC-derived MVs in an *E. coli* pneumonia mouse model resulted in a higher bacterial clearance, which was in part due to the increased monocyte phagocytosis. Moreover, survival improved partly through KGF secretion. The results also showed that the pre-stimulation of MSCs with a Toll like Receptor-3 (TLR-3) agonist could lead to the release of more effective MVs and further enhancement of bacteria’s monocyte phagocytosis. It has been revealed that the binding and uptake of MSC-MVs into human monocytes and injured alveolar epithelial cells were mediated *via* the CD44 receptor on the mentioned target cells, which was necessary for their therapeutic effects. In addition, MVs enhanced intracellular ATP levels in injured alveolar epithelial cells and reduced the secretion of inflammatory cytokines including Tumor Necrosis Factor-alpha (TNF-α) in human monocytes, suggesting the metabolomics and immunomodulatory effects of MVs derived from MSCs. Interestingly, MSC-MVs expressed Cyclooxygenase2 (COX2) mRNA. COX2 is the key enzyme in Prostaglandin E2 (PGE2) synthesis that is an essential factor for transforming the polarization of monocyte-macrophage M1 into M2 phenotype. It was suggested that the increment in PGE2 secretion by monocytes following the transfer of COX2 mRNA from MSC-MVs to these cells caused the phenotype switch toward an anti-inflammatory state. Thus, it could be suggested that MSC-MVs mitigated lung inflammation, cytokine permeability, and bacterial growth and improved survival directly through KGF transfer or indirectly *via* activating monocytes. This therapeutic effect of MVs was abrogated by KFG neutralizing antibody, proposing a possible mechanism for the anti-bacterial effect of MSC-MVs (Monsel et al., 2015; Abreu et al., 2016). Since the anti-bacterial effect of KGF was previously reported in MSCs (Lee et al., 2013), these studies supported the hypothesis that MVs conserve the anti-microbial effects of parent cells partly through their growth factors content including KGF.

In addition to the beneficial effects of stem cell-Exo in a mouse model of ALI, Park et al. (2019) recently evaluated the therapeutic effects of BM-MSC-MVs on *ex vivo* perfused human lungs with severe pneumonia induced by *Escherichia coli* that resulted in the significant enhancement of alveolar fluid clearance, reduction of lung protein permeability leading to lower bacterial load, and decrement of the median pulmonary artery pressure. The anti-microbial activity of human BM-MSC-MVs could be further increased by the pre-treatment of MSCs with a TLR-3 agonist, Poly (I:C), before the isolation of MVs, which led to lower neutrophils infiltration in the injured lung. Additionally, isolated human alveolar macrophages increased anti-microbial activity with MSC-MVs treatment *in vitro*, which resulted in the enhancement of bacterial clearance in the injured lung (Park et al., 2019; Al-Khawaga and Abdelalim, 2020). A noteworthy point in the studies carried out by Monsel et al. (2015) and Park et al. (2019) was the preconditioning of MSCs with a TLR-3 agonist, Poly (I:C). Further studies showed that the pre-stimulation of parent MSCs with poly (I:C) could increase the anti-microbial and immunomodulatory proteomic profile of EVs (Mayo et al., 2019; Pierce and Kurata, 2021). They also indicated various AMPs in MSC-EVs including dermcidin, lactoferrin, lipocalin 1, lysozyme C, neutrophil defensin 1, S100A7 (psoriasin), S100A8/A9 (calprotectin), and histone H4. Several AMPs helped fight against various bacteria, fungi, and viruses (Pierce and Kurata, 2021). However, these AMPs remained unaltered by poly (I:C) pre-stimulation. Up to now, no study has been performed on the exact effect of AMPs through transfer with stem cell-Exo, which requires special attention in future. Furthermore, it should be noted that although many studies have dealt with MVs based on the separation method (Zhu et al., 2014; Monsel et al., 2015; Park et al., 2019), they have actually isolated a combination of MVs and exosomes.

Immunomodulatory and immunostimulatory properties of stem cell-Exo partly depend on functional miRNAs by exosomes. Hao et al. (2019) investigated the effects of human MSC-Exo on *Escherichia coli* pneumonia-induced acute lung injury in C57BL/6 mice. They found that exosomes administration was associated with high levels of Leukotriene (LT) B4 and improvement of bacteria clearance in the injured alveolus. It has been found that LTB4 augmented phagocytosis and the release of anti-microbial agents and increased host defense against pneumonia and sepsis. Production of LTB4 was suppressed by an ATP-binding cassette transporter called Multidrug Resistance–Associated Protein 1 (MRP1). The underlying mechanism of the anti-microbial activity of MSC-Exo was through the inhibition of MRP1 expression partly *via* the transfer of miR-145, which resulted in increased LTB4 production that led to the enhancement of bacterial phagocytosis through LTB4/BLT1 signaling. Previous studies indicated that miR-145 was one of the top 10 most abundant miRNAs detected in MSCs and MSC-Exo, which could directly inhibit MRP1 expression in breast and gallbladder cancers (Hao et al., 2019).

The use of stem cell-Exo for reducing the complications caused by bacteria seems to be attractive. Sepsis is known as a serious and life-threatening condition with high morbidity and mortality, which increases when the host body’s response to infections including bacterial infections causes injury to its own organs (Cheng et al., 2020). Interleukin-1b (IL-1b), as a serious pro-inflammatory cytokine, increases in the early stage of sepsis and is involved in the severity and evolution of organ dysfunction. In the study conducted by Song et al. (2017) BM-MSCs were pre-stimulated by IL-1b prior to the isolation of exosomes. Then, the effect of these exosomes was investigated in a cecal ligation and puncture-induced mouse model of sepsis. The results showed that IL-1b enhanced the therapeutic effect of MSCs-Exo against sepsis by inducing macrophage polarization to an anti-inflammatory M2 phenotype (Song et al., 2017). The results also revealed that exosomes derived from MSCs contained high levels of miR-146a, which is a well-known anti-inflammatory microRNA. Transfer of miR-146a by exosomes to recipient macrophages regulated M1-M2 transition, reduced inflammation, and enhanced survival in septic mice. In addition, transfection of miR-146a inhibitors partially abrogated the immunomodulatory properties of exosomes. Overall, IL-1b pre-stimulation effectively increased the immuno-modulatory properties of MSCs partially through the exosome-mediated transfer of miR-146a (Song et al., 2017; Cheng et al., 2020). All in all, exosomes derived from stem cells had the anti-bacterial capacity against intracellular bacterial infections. It could be a proof-of-principle that therapeutic approaches based on exosomes derived from MSCs offer a promising path forward.

Research on the administration of exosomes for drug delivery, particularly antibiotics, is still in its initial steps. In the study carried out by Yang et al. (2018) exosomes were isolated form RAW 264.7 mice macrophages and were incubated with an antibiotic agent called linezolid. They evaluated the effect of linezolid-exosomes on Methicillin-Resistant *Staphylococcus aureus* (MRSA)-infected macrophages in a mouse model of MRSA. Briefly, *Staphylococcus aureus* lives inside phagocytes and is a strain with antibiotic resistance, which can lead to sepsis, infective endocarditis, osteomyelitis, and necrotizing pneumonia. The results showed that the delivery of exosome-encapsulated antibiotics was more effective against intracellular MRSA infections compared to free linezolid antibiotics (Yang et al., 2018). In this regard, exosomes derived from stem cells and immune cells had the capacity for delivery of anti-microbial agents against intracellular pathogen infections. Yet, further studies are required for clinical applications.

## The Application of Stem Cell-Derived Exosomes in Viral Diseases

Some contents in exosomes derived from stem cells can play substantial anti-viral roles by inhibiting viral replication and inducing immune responses (Longatti, 2015). Clinical trials have shown that the exosomes released from different cells can be novel therapeutic strategies against viruses including hepatitis, HIV, and COVID-19. To the best of our knowledge, there are limited studies regarding the application of stem cell-Exo in viral diseases. Qian et al. (2016) investigated the effects of the secreted exosomes from umbilical-MSCs on hepatitis C virus infection *in vitro*. The results indicated that a profile of miRNAs in the exosomes including let-7f, miR-145, miR-199a, and miR-221 was involved in the direct suppression of the RNA replication of hepatitis C virus (Qian et al., 2016).

Exosomes also play important roles in the interplay between the virus and various immune cells in hepatitis viruses. In particular, virus-infected cells release exosomes that affect the host immune system. An *in vitro* study on hepatitis C virus infection showed that macrophages’ exosomes contained miR-29 family members that exerted anti-viral effects on Huh7 cells (Zhou et al., 2016). Kouwaki et al. (2016) also reported that hepatitis B virus-infected hepatocytes released exosomes containing viral nucleic acid, which activated the innate immune response. They found that the microRNA levels of miR-21 and miR-29a increased in the exosomes of the infected hepatocytes that stimulated macrophages (Kouwaki et al., 2016). Furthermore, the previous studies emphasized that miR-21 was enriched in exosomes derived from BM-MSCs (Shi et al., 2018) and human umbilical cord MSCs (Chen et al., 2020). The presence of miR-29a was detected in BM-MSCs, as well (Lu et al., 2020; Tan et al., 2020). Therefore, the exosomes derived from such sources may be effective against hepatitis viruses.

Sims et al. (2014) described the role of neural stem cell-Exo in cellular viral entry. The findings showed that the exosomes contained T-cell immunoglobulin mucin protein 4, which acted as a phosphatidylserine receptor and mediated adenovirus type 5 entries. Clarifying the virus/exosome pathways and exosome trafficking may provide a potentially therapeutic option (Sims et al., 2014).

Recent studies have proposed the anti-HIV activity of exosomes (Yong et al., 2018). It has been conducted on the application of exosome-containing miRNAs in the treatment of HIV (Yong et al., 2018). In this context, a variety of miRNAs including miR-28, miR-150, miR-223, miR-382 (Wang et al., 2009), miR-29a, miR-29b, miR-149, miR-324, miR-378 (Hariharan et al., 2005), miR-125b (Mantri et al., 2012), and miR-198 (Sung and Rice, 2009) have been explored in the host exosomes involved in HIV therapy (Madison and Okeoma, 2015). Several studies proposed the presence of the mentioned miRANs in the exosomes derived from different sources of stem cells. Accordingly, the exosomes derived from BM-MSCs contained miR-29a (Lu et al., 2020; Tan et al., 2020), miR-150 (Qiu et al., 2021; Wu et al., 2021), miR-223 (Chen L et al., 2018b), miR-29a (Su et al., 2019), and miR-125b (Wang et al., 2019). miR-223 was also identified in the exosomes derived from umbilical cord MSCs (Wei et al., 2020; Liu et al., 2021). These findings indicated that MSCs could be considered in anti-HIV strategies. Recent studies on the treatment of viruses also indicated the potentiality of exosomes for encapsulating bioactive molecules in drug delivery systems. Therefore, transferring anti-HIV RNAs through artificial exosomes or exosomes derived from stem cells may be promising in the HIV treatment.

Several studies have demonstrated the beneficial effects of exosomes from stem cells on the treatment of respiratory viruses (Popowski et al., 2021). During the COVID-19 pandemic, researchers focused on the application of stem cell-Exo, as a treatment option. Considering the beneficial effects of stem cell-Exo on the management of cytokine storm, tissue repair, and viral suppression, exosomes may be considered a promising therapeutic option. A prospective non-randomized open-label cohort study proposed the safety and efficacy of exosomes derived from allogeneic BM-MSCs in severe COVID-19. This study revealed that these exosomes attenuated cytokine storm, recovered oxygenation, and improved immunity cell counts (Sengupta et al., 2020). Generally, the Receptor-Binding Domain (RBD) of the SARS-CoV-2 spike protein recognizes the Angiotensin-Converting Enzyme 2 (ACE2) receptor to enter host cells. The application of exosomes that effectively bind to SARS-CoV-2 may prevent the virus from entering the cells. Interestingly, a previous study revealed that the exosomes expressing ACE2 dose-dependently prevented the binding of the RBD of the SARS-CoV-2 spike protein to ACE2 + cells (El-Shennawy et al., 2020). Hence, engineering of stem cell-Exo to overexpress ACE2 may competitively block the binding of SARS-CoV-2 to ACE2-expressing cells (Inal, 2020). In addition, stem cell-Exo contributed to organ regeneration and repair (Basu and Ludlow, 2016; Maqsood et al., 2020). Therefore, the tissue and organ destruction occurring in COVID-19 may be improved by using exosomes. Suppression of cytokine storm is yet another issue in COVID-19 management. The immunomodulatory function of MSC-Exo has made them a potential therapeutic option for cytokine storm. These exosomes decrease proinflammatory cytokines (Liang et al., 2020), inhibit CD4^+^ and CD8^+^ T cells (Taechangam et al., 2019), reduce the proliferation and activation of NK cells (Moloudizargari et al., 2021), and improve the release of IL-4, IL-10, and TGF-β (Jayaramayya et al., 2020). Based on these pieces of evidence, the anti-viral properties of the exosomes released from stem cells are related to their key molecules. These molecules may disturb the virus survival or inhibit the side effects caused by them.

## The Application of Stem Cell-Derived Exosomes in Fungal and Parasitic Diseases

Similar to the studies performed on viruses, limited data are available for identifying the effects of stem cell-Exo on human fungal infections. Only one study conducted by Cruz et al. (2015) demonstrated that the systemic administration of exosomes derived from human BM-MSCs improved allergic airway inflammation induced by *Aspergillus* hyphal extract in an immunocompetent mouse model of severe refractory neutrophilic asthma (Cruz et al., 2015). Hence, research on the impact of stem cell-Exo on fungal diseases is still in its infancy, and further investigations are necessary.

Despite of distinct properties of stem cell-Exo, no evidence is available regarding the therapeutic application of stem cell-Exo in the context of parasite infections. There are more than 1 billion cases of parasitic diseases in the world including malaria (Murray et al., 2014) and neglected tropical diseases such as helminthiases, Chagas disease, and leishmaniosis (Coakley et al., 2015), with an increasing prevalence in developing regions such as Eastern Asia, Sub-Saharan Africa, and the Americas (Lustigman et al., 2012). Hence, attention has to be paid to the potential of exosomes as a biomarker and therapeutic agent in parasite diseases.

## Advantages and Challenges in the Use of Stem Cell-Derived Exosomes

Application of stem cells in the treatment of different human diseases, especially microbial infections, has shown effective outcomes. Nevertheless, there are still safety concerns like lower survival after transplantation as well as the possibility of pulmonary embolism, tumorginicity, and uncontrolled differentiation. Yet, stem cell-Exo are highly stable due to biolayer lipid membrane, small size, low immunogenicity, easy storage at −80°C for a long time without toxic agents, and easier procedure for delivery and management (Keshtkar et al., 2018). Like parent cells, exosomes have immunomodulatory and immunosuppressive properties that enable them to participate in various disease models. Thus, stem cell-Exo represent an alternative to stem cell therapies, with no safety issues regarding regenerative medicine.

Mesenchymal Stem Cell-derived Exosomes have been reported to decrease the side effects of cell therapy (Malekpour et al., 2021). Therefore, they can be a good platform for various applications in the treatment of various diseases. For instance, manipulation of exosomes by loading therapeutic compounds as well as transferring interfering RNA, miRNA, and oligonucleotides enhances their efficiency (Zhang et al., 2021). In the study by Melzer et al. (2019) evaluated the loading of compound paclitaxel into MSC-Exo. They showed that manipulated MSC-Exo notably decreased the breast tumor volume and suppressed the metastasis compared to MSC-Exo alone (Melzer et al., 2019). The biocompatibility potential of exosomes also makes them an ideal candidate for drug delivery like antibiotics (Huang and Lai, 2019). In the study carried out by Yang et al. (2018) exosomes were isolated form RAW 264.7 mice macrophages and were incubated with an antibiotic agent called linezolid. They evaluated the effect of linezolid-exosomes on Methicillin-Resistant *Staphylococcus aureus* (MRSA)-infected macrophages in a mouse model of MRSA. Briefly, *S. aureus* lives inside phagocytes and is a strain with antibiotic resistance, which can lead to sepsis, infective endocarditis, osteomyelitis, and necrotizing pneumonia. The results showed that the delivery of exosome-encapsulated antibiotics was more effective against intracellular MRSA infections compared to free linezolid antibiotics (Yang et al., 2018). Hence, they have been nominated as ideal vehicles for therapeutic applications. Despite the aforementioned advantages, research on development and treatment based on stem cell-Exo is still in its infancy. There are also some hurdles that must be overcome prior to translation from bench to bed. These include the lack of standard isolation and purification methods for exosomes, lack of complete information about the exact cargos of these vesicles, and existence of heterogeneity in released vesicles as a result of physiological changes in the cells’ extracellular space (Huang and Lai, 2019; Brakhage et al., 2021).

## Conclusion

Extracellular Vesicles, especially exosomes, secreted by stem cells have the same anti-microbial potential and immunomodulatory ability as their parent cells. Hence, clinical applications of stem cell-Exo can possibly overcome the shortage of stem cells for the treatment of microbial and other infectious diseases and, at the same time, affect the field of novel medicine from cellular to acellular therapy. Both intact and engineered exosomes have been applied and their therapeutic effects on various infectious diseases have been demonstrated in preclinical studies and limited clinical trials. Exosomes perform a part of their antimicrobial activity through the direct transfer of mRNA, miRNA, and protein cargos, while their beneficial effects are mostly applied indirectly through the reprogramming of immune cells and the activation of innate and adaptive immune responses.

Although the underlying mechanism of stem cell-Exo has not been specified exactly and completely, the anti-microbial activity of exosomes appears to be more indirect than direct.

Moreover, many barriers are still needed to be eliminated prior to the application of stem cell-Exo as anti-microbial agents in clinical settings.

## Author Contributions

All authors took part in literature search, manuscript preparation, and editing.

## Conflict of Interest

The authors declare that the research was conducted in the absence of any commercial or financial relationships that could be construed as a potential conflict of interest.

## Publisher’s Note

All claims expressed in this article are solely those of the authors and do not necessarily represent those of their affiliated organizations, or those of the publisher, the editors and the reviewers. Any product that may be evaluated in this article, or claim that may be made by its manufacturer, is not guaranteed or endorsed by the publisher.
